# Polychromatic digital holographic microscopy: a quasicoherent-noise-free imaging technique to explore the connectivity of living neuronal networks

**DOI:** 10.1117/1.NPh.7.4.040501

**Published:** 2020-10-16

**Authors:** Céline Larivière-Loiselle, Erik Bélanger, Pierre Marquet

**Affiliations:** a Université Laval, Centre de recherche CERVO, Québec, Canada; b Université Laval, Département de physique, de génie physique et d’optique, Faculté des sciences et de génie, Québec, Canada; c Université Laval, Centre d’optique, photonique et laser, Québec, Canada; d Université Laval, Département de psychiatrie et neurosciences, Faculté de médecine, Québec, Canada

**Keywords:** digital holographic microscopy, coherent noise, denoising, neurons, axons, dendrites

## Abstract

**Significance:** Over the past decade, laser-based digital holographic microscopy (DHM), an important approach in the field of quantitative-phase imaging techniques, has become a significant label-free modality for live-cell imaging and used particularly in cellular neuroscience. However, coherent noise remains a major drawback for DHM, significantly limiting the possibility to visualize neuronal processes and precluding important studies on neuronal connectivity.

**Aim**: The goal is to develop a DHM technique able to sharply visualize thin neuronal processes.

**Approach**: By combining a wavelength-tunable light source with the advantages of hologram numerical reconstruction of DHM, an approach called polychromatic DHM (P-DHM), providing OPD images with drastically decreased coherent noise, was developed.

**Results**: When applied to cultured neuronal networks with an air microscope objective (20×, 0.8 NA), P-DHM shows a coherent noise level typically corresponding to 1 nm at the single-pixel scale, in agreement with the $1/\sqrt{N}$-law, allowing to readily visualize the 1-μm-wide thin neuronal processes with a signal-to-noise ratio of ∼5.

**Conclusions**: Therefore, P-DHM represents a very promising label-free technique to study neuronal connectivity and its development, including neurite outgrowth, elongation, and branching.

## 1 Introduction

Digital holographic microscopy (DHM), an important approach in the field of quantitative-phase imaging techniques, has started to be efficiently used in the last decade as a label-free, high-resolution, and live-imaging method for quasitransparent biological samples such as living cells.^1^^–^^3^ Due to its quantitative phase signal (QPS), it provides accurate and noninvasive measurements of various important cellular parameters, including dry mass,^4^ whole-cell stiffness,^5^ membrane fluctuations,^6^ transmembrane water fluxes,^7^ and absolute cell volume.^8^ Due to its aforementioned advantageous imaging characteristics, DHM has been successfully used in cellular neuroscience revealing that significant transmembrane water movements are associated with neuronal activity.^7^^,^^9^ However, coherent noise (an acknowledged source of image quality limitation in coherent optical microscopy systems^10^) remains an issue, particularly when considering an off-axis DHM configuration,^11^ as it requires the use of a highly or partially coherent light source.^12^ Seen as granularity in the quantitative-phase (QP) images, it is caused by unwanted reflections and small defects or dirt in the optical path. This precludes the possibility to explore minute cellular structures especially neuronal processes, a major part of neural circuits through which the information is integrated and conveyed between neurons. Typical coherent noise amplitude is larger than the QPS generated by a significant part of the neuronal processes, especially the thin projections including dendrites.

Although it is far beyond the scope of this letter to summarize all strategies for reducing or mitigating coherent noise in DHM, they can be roughly divided into two main categories. The first relies on numerical approaches using algorithms to reduce coherent noise.^13^^–^^17^ To date, applications using these techniques have mostly focused on the reduction of coherent noise of large-size real-world objects. Some of these approaches have reported impressive denoising capability by allowing the removal of up to 98% of the coherent noise, while preserving the image contrast. The second strategy is based on optical instrumentation development.^18^^,^^19^ As far as QP images in a microscopy setting are concerned, efficient coherent noise reduction approaches based on either object or camera motion, or on the use of different laser modes have been developed.^20^^–^^22^ Specifically, in a transmission configuration aimed at exploring biological cells, a lateral shift of the camera^21^ significantly reduced the coherent noise, while a noise reduction approaching the ideal curve of $1/\sqrt{N}$ was obtained by slightly moving the object.^20^ A strategy using different laser modes represents an interesting alternative to significantly reduce coherent noise of quantitative-phase images^22^ while avoiding mechanical motion. Elsewhere, Kemper et al.^23^ used the reduced coherence length of light-emitting diodes, compared to traditional gas lasers, to lessen the coherent noise of QP images dedicated to exploring surface topography. From this idea, Kosmeier et al.^24^ decreased the coherent noise of QP images of living cancer cells by emulating a broadband light source of 30 nm and numerically superimposing reconstructed object waves from a set of different wavelengths.

Following preliminary observations suggesting that the coherent noise of optical path difference (OPD) images can be reduced by taking advantage of a multispectral hologram recording,^25^ we present in this letter a multiwavelength off-axis DHM, in a transmission configuration, providing quasicoherent-noise-free OPD images. Our specific approach, called polychromatic DHM (P-DHM), has been developed by reconstructing each OPD image from its corresponding hologram recording at a specific wavelength in an aberration-free manner due to a flexible numerical approach based on object wave propagation. The averaged OPD image corresponds to the numerical reconstruction of 36 holograms recorded over a wide range of wavelengths from the visible to the near-infrared. This averaging exhibits a noise level revealing a set of thin neuronal processes within living cultured neural networks normally undetectable with off-axis DHM.

## 2 Materials and Methods

### 2.1 Polychromatic Digital Holographic Microscope

**Fig. 1:**
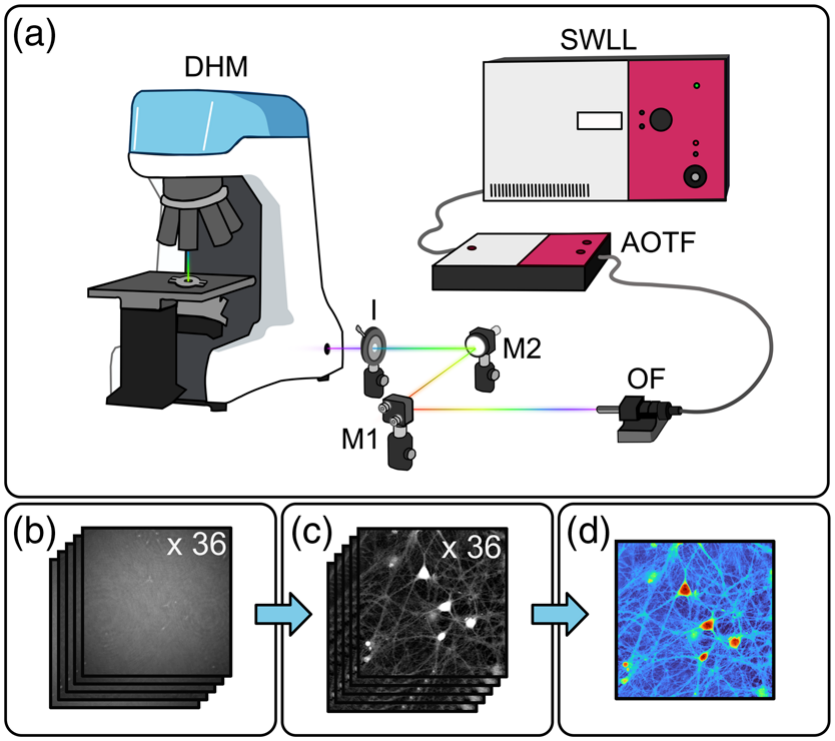
(a) Experimental setup injecting the SWLL and AOTF into the DHM using an OF, two mirrors (M1 and M2), and an iris (I). (b) Stack of holograms used to obtain (c) a stack of OPD images which are averaged to obtain (d) the P-DHM image.

The polychromatic setup was assembled from a commercial transmission Mach–Zehnder interferometer-based DHM (DHM T-1003, Lyncée Tec), described previously.^26^^,^^27^ The genuine internal coherent light source of the DHM was bypassed by a tunable wavelength laser system, consisting of a supercontinuum white-light laser (SWLL) (SuperK EXR-20, NKT Photonics) coupled with a multiwavelength acousto-optic tunable filter (AOTF) (SuperK SELECT VIS-nIR, NKT Photonics), injected in the microscope with a delivery optical fiber (OF) (FD7-PM, NKT Photonics), as shown in Fig. 1(a). The DHM setup works by splitting a coherent light source into two: the object beam, which passes through the sample, and the reference beam. The microscope objective (MO), located in the object arm, has a magnification of 20× with a free working distance of 400  μm and an NA of 0.80 (HC PL APO, Leica). At the exit of the interferometer, the two beams interfere producing a hologram in the camera plane. The setup has an off-axis geometry, meaning that a small angle is introduced between the object and reference waves.^28^ A digital camera equipped with a monochrome 2.3-megapixel Sony IMX174 CMOS sensor with a pixel size of 5.86  μm (acA1920, Basler) captured the holograms, which were integrated over a period varying between 200  μs up to 14 ms, depending on the operating wavelength.

### 2.2 Methodology

The methodology begins with the manual acquisition of a stack of 36 holograms recorded sequentially at wavelengths ranging from 500 to 850 nm, in steps of 10 nm, as shown in Fig. 1(b). For each hologram at each wavelength: the exposure time was adjusted to optimize the use of the dynamic range of the camera and the holograms were numerically reconstructed with numerical refocusing to obtain QP images by simulating the illumination with a digital reference wave.^28^ The hologram numerical reconstruction makes it possible to correct at each wavelength the aberrations of the object wavefront,^29^^–^^32^ a fundamental step to obtain quality coherent-noise-reduced images. The exposure time adjustment, numerical refocusing, and aberrations compensation procedures are described in more detail in the Supplementary Material. The QP images thus reconstructed provide a QPS, corresponding to the phase delay induced by the observed quasitransparent cells. At a given wavelength λ, for each of the (m,n)-pixel coordinates, the QPS is given by

$$\Delta \varphi (m,n)=\frac{2\pi }{\lambda }\Delta n(m,n)h(m,n),$$ (1)

where Δn(m,n) is the difference between the refractive indices of the cell and the extracellular medium, and h(m,n) is the cell height. The following step makes the QP images wavelength independent with the aim of performing OPD frame averaging. For each wavelength $\lambda_{i}$, OPD images are calculated according to the following relationship:

$${\mathrm{OPD}}_{i}(m,n)=\frac{{\Delta \varphi }_{i}(m,n)\lambda_{i}}{2\pi }.$$ (2)

The multiwavelength stack of 36 OPD images, as shown in Fig. 1(c), are then averaged pixelwise to form a mean OPD image according to

$$\langle \mathrm{OPD}(m,n)\rangle =\frac{1}{N}\overset{N}{\underset{i=1}{\sum }}{\mathrm{OPD}}_{i}(m,n),$$ (3)

where N=36 is the number of holograms. This image, as shown in Fig 1(d), is the P-DHM denoised image. As an experimental control, another stack of 36 holograms was all captured at the same wavelength, namely, 540 nm. Then, a monowavelength OPD averaged image (the control image) is obtained through the same methodology as described for P-DHM.

## 3 Results and Discussion

### 3.1 Polychromatic Digital Holographic Microscopy Denoising

**Fig. 2:**
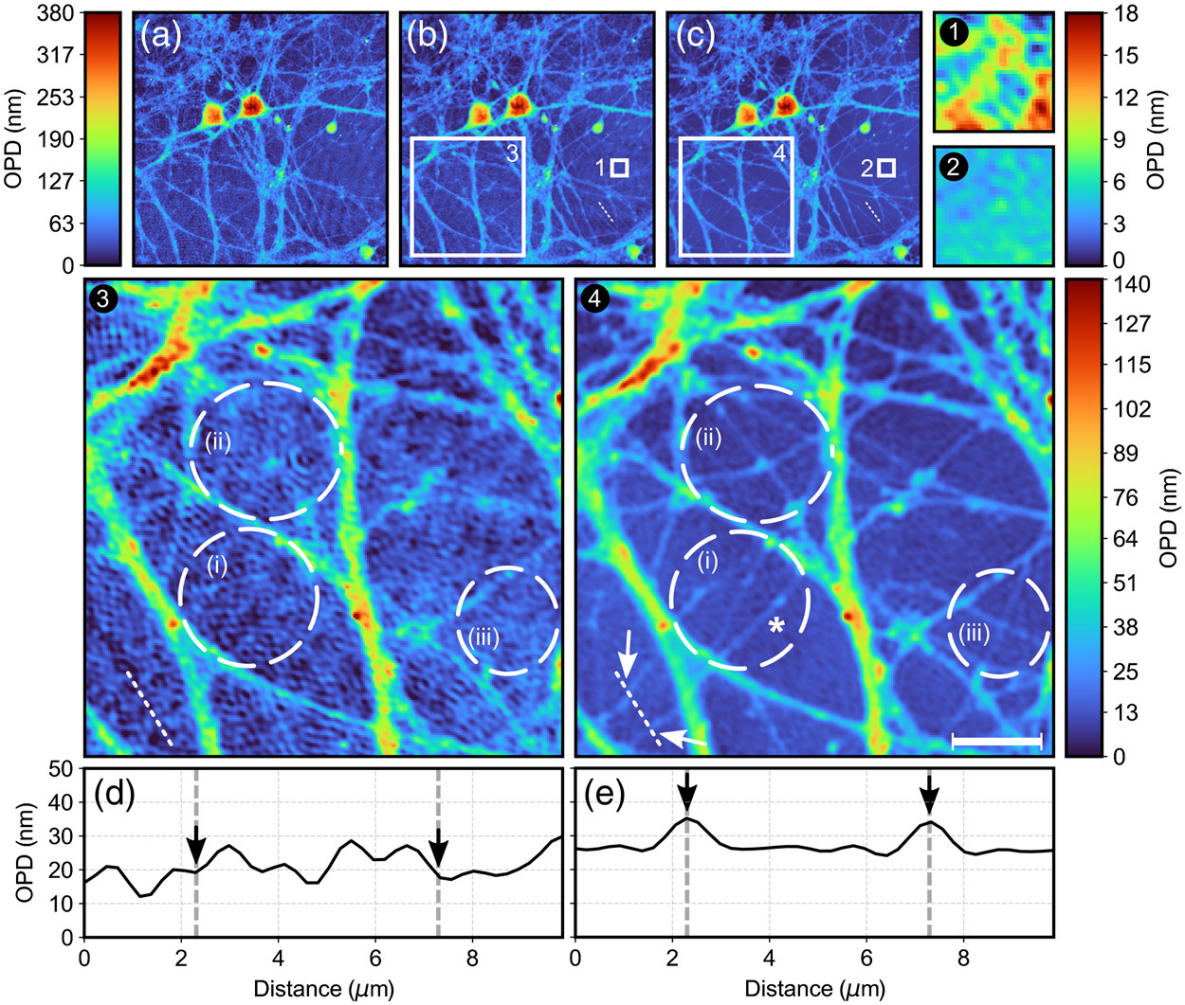
(a) Single-shot image acquired with 540-nm illumination, (b) control image, and (c) P-DHM image. The white dotted lines in (b) and (c) represent line profiles further analyzed in Fig. 3. (1) and (2) Background section of the control and P-DHM images, respectively. (3) and (4) Neuronal process area of the control and P-DHM images, respectively. (i)–(iii) The white dashed circles highlight cell features and the asterisk identifies a small nerve extension further analyzed in Fig. 4. (d) and (e) Height profiles extracted from the white dotted lines in the bottom left corner of (3) and (4). Scale bar indicates 10  μm.

Figure 2 highlights the denoising capability of the proposed approach by showing two averaged OPD images of the same field-of-view of a rat neuronal cell culture—one corresponds to the control image and the other to P-DHM. For each image group, the color bar is globally rescaled to properly appreciate the denoising outcome. A typical OPD image, obtained from a single-shot QP image at 540 nm using Eq. (2), is shown in Fig. 2(a), while Figs. 2(b) and 2(c) show the control and P-DHM images, respectively. The reduced granular noise pattern is clearly visible, especially in the background as shown in Figs. 2(b1) and 2(c2). This coherent noise reduction is also particularly noticeable in Figs. S1(a)–S1(c) in the Supplementary Material showing 3D rendered images with the OPD as vertical axis. In contrast, the images in Figs. 2(b3) and 2(c4) focus on an area rich in neuronal processes and the dashed white circles labeled (i)–(iii) draw attention to features unveiled solely by P-DHM. While the “X” shape formed by neuronal processes highlighted in (ii) can hardly be identified in the control image, it is sharply visible in the P-DHM image. In addition, a set of thin neuronal processes [emphasized in (i) and (iii) areas] are revealed by P-DHM but are not visible in the control image. These cell structures, taking the form of fine intersecting neuronal processes, are clearly visible in Figs. S1(b3) and S1(c4) in the Supplementary Material. Figures 2(d) and 2(e) show the individual OPD profiles extracted from the control [Fig. 2(b3)] and P-DHM images [Fig. 2(c4)], respectively, at the position indicated by the dotted white line. The OPD spatial fluctuations along the profile corresponding to the control image [Fig. 2(d)] preclude the possibility to detect the presence of the two thin neuronal processes, which have in contrast a clear signature in the form of two peaks [black arrows, Fig. 2(e) and white arrows, Fig. 2(c4)] along the P-DHM profile. This results from the fact that the background spatial fluctuations (exhibiting amplitudes comparable to those of the two peaks and mainly reflecting the presence of coherent noise) have been drastically reduced. For this proof-of-principle, the 36-hologram acquisition time was around 10 min reflecting, however, a manual approach, requiring performing several tens of clicks to operate both the software of the microscope and laser. An automated approach could easily achieve an acquisition time under a second.

**Fig. 3:**
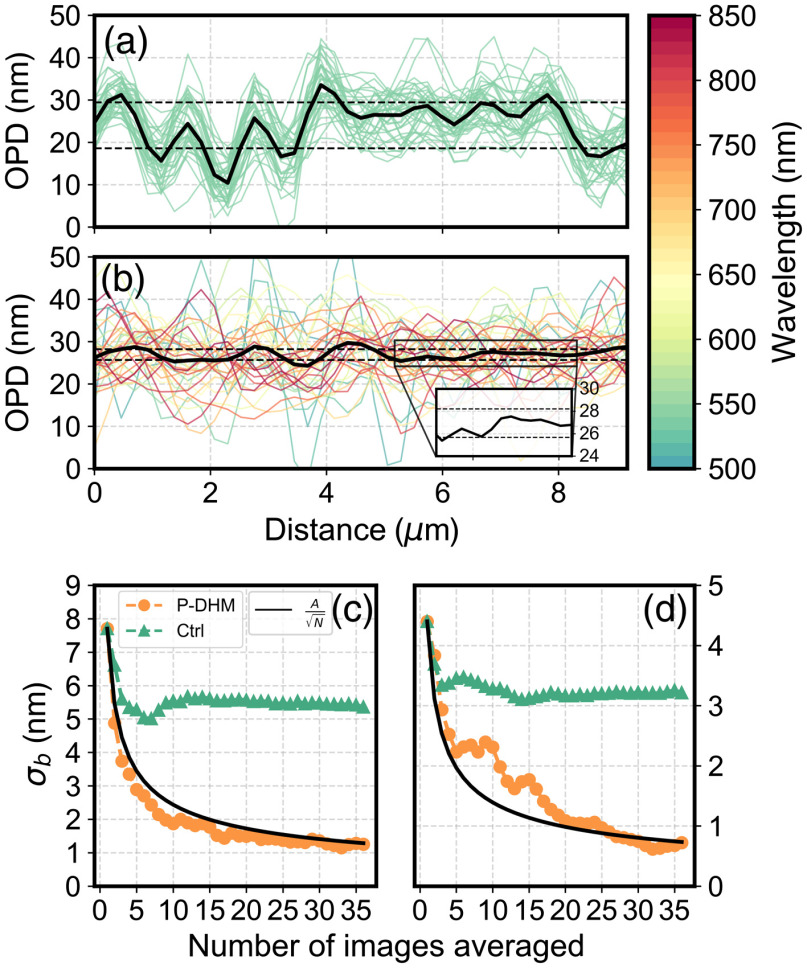
Analysis of the height profile from Figs. 2(b) and 2(c). The mean profiles of (a) the control and (b) P-DHM are shown in black, and the single-shot images used for the frame averaging are displayed in color, corresponding to the wavelength used for acquisition. Black dotted lines correspond to the $\sigma_{b}$ of each specific profile, in this case 5.4 and 1.2 nm for the control case and P-DHM, respectively. The inset in (b) is an enlarged portion of the line profile. (c) $\sigma_{b}$ of the line profile for the (a) control case in green and (b) P-DHM in orange as a function of N. The black line is the $1/\sqrt{N}$-curve with A chosen as the first data point. (d) $\sigma_{b}$ of another line profile extracted from a different location in the same neuron image, displaying a different behavior as a function of N.

**Fig. 4:**
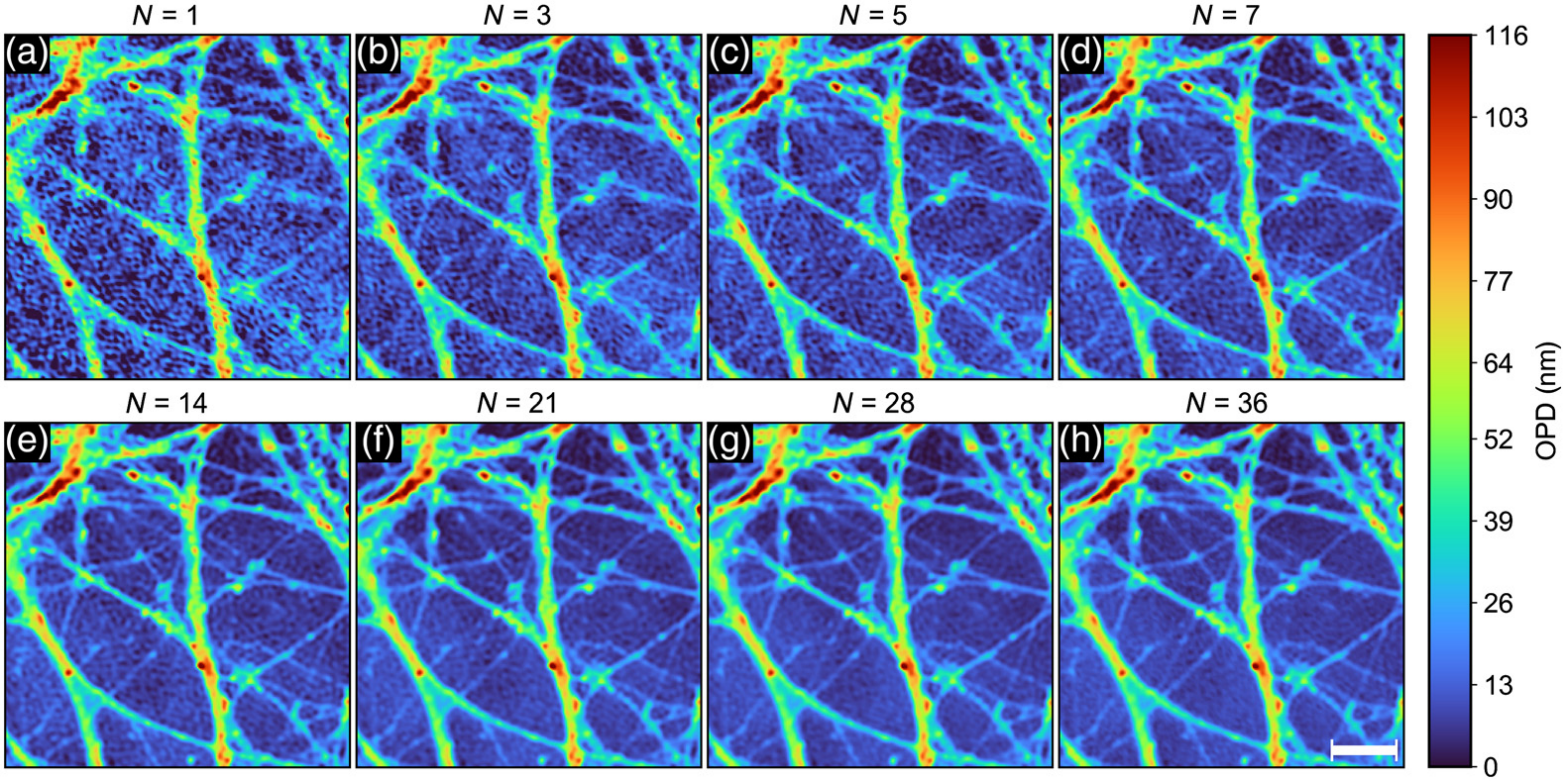
P-DHM image as N increases from (a) N=1 to (h) N=36. Scale bar indicates 10  μm.

### 3.2 Polychromatic Digital Holographic Microscopy Denoising Quantification

To better understand coherent noise reduction, Figs. 3(a) and 3(b) show both the monowavelength average and P-DHM OPD profiles corresponding to a linear height profile within the background [dotted white lines, Figs. 2(b) and 2(c)] accompanied in each case by the 36 individual OPD background profiles that were used to calculate these two average profiles. The values of standard deviations of both background average profiles $\sigma_{b}$, indicated by dashed black lines in Figs. 3(a) and 3(b), provide a quantification of the drastic reduction in coherent noise achieved by P-DHM. Conversely, the 36 individual background profiles of the control case [Fig. 3(a)] are remarkably similar to each other while those of P-DHM [Fig. 3(b)] (each of them corresponding to a specific wavelength) are very different from each other. This illustrates the fact that the change of wavelength is likely to significantly decorrelate the coherent noise between each of the OPD images, therefore explaining why the OPD frame averaging procedure in P-DHM is highly effective in reducing coherent noise.

A set of more than 50 OPD line profiles randomly selected in the background have been analyzed and Figs. 3(c) and 3(d) show the two most representative $\sigma_{b}(N)$ of this OPD line profile set for both the control case and P-DHM. Notably, regarding P-DHM, $\sigma_{b}(N)$ [despite a behavior that can be erratic for values of N≤15; Fig. 3(d)] always accurately follow a $1/\sqrt{N}$-decrease [Fig. 3(c), resulting from the line profile of Fig. 3(b)]. In contrast, the monowavelength case has a noise reduction curve exhibiting a much smaller decrease, stabilizing already after the averaging of five images. Based on these 50 OPD profiles, an average background standard deviation of ${\overset{¯}{\sigma }}_{b,540}=5.7\pm 1.4\;\mathrm{nm}$, ${\overset{¯}{\sigma }}_{b,\mathrm{ctrl}}=4.1\pm 0.9\;\mathrm{nm}$, and ${\overset{¯}{\sigma }}_{b,P\text{-}\mathrm{DHM}}=1\pm 0.3\;\mathrm{nm}$, respectively, for the single-shot OPD image at 540 nm, the control case, and P-DHM is obtained. These results demonstrate a noise reduction by a factor of ∼1.4 for the control case and by a factor close to 6 for the P-DHM when using 36 OPD images. Considering 1-μm-wide thin neuronal processes (corresponding to ∼3 to 4 pixels with the 0.8-NA MO of 20× magnification) and having a characteristic OPD of 1 to 9 nm, this results in a signal-to-noise ratio (SNR) of ∼5 at the single-pixel level. As for the mid-size neuronal extensions and larger axons, single-pixel SNRs of 20 to 75 are attained. This approach was also tested on other cell types, including fibroblasts, and a similar advantage in terms of denoising was systematically observed.

Interestingly, an examination of the increasingly denoised P-DHM image as a function of the number of averaged images (ascending order of wavelengths) as shown in Fig. 4 reveals that the section of the thin neuronal process identified by an asterisk at the bottom of circle (i) in Fig. 2(c4) requires at least a three-image average to become apparent (as a dotted line) to the naked eye, and five images for its full length to emerge clearly.

### 3.3 Dispersion of the Observed Cells

According to Eqs. (1)–(3), averaging OPD images acquired at different wavelengths will provide an accurate averaged OPD image if the dispersion of the observed cells does not significantly differ from that of the surrounding medium, i.e., water. In this situation, the values of the OPD generated by the observed cell must remain constant whatever the considered wavelength. The results of Fig. S2 in the Supplementary Material (showing the OPD measurements as a function of the wavelength for the left-hand side neuronal cell body in Fig. 2) support this fact. This is an expected result, previously demonstrated by Rappaz et al.,^33^ and remains valid for mammalian nonpigmented cells usually composed of ∼80% water and for which the refractive index dispersion specifically due to their constituents can be neglected.

## 4 Conclusion

The P-DHM technique presented in this letter, achieved with a series of 36 digital holograms recorded at regular intervals within a wavelength range between 500 and 850 nm, using a tunable wavelength laser system, enables us to obtain OPD images with a coherent noise reduced as much as possible. Specifically, a multistep numerical reconstruction of each digital hologram, allowing a wavelength-specific aberration correction, results in a series of corrected OPD images displaying uncorrelated coherent noise patterns. The averaging of 36 images provides an OPD image with a coherent noise reduced globally by a factor of 6 in agreement with the ideal curve of $1/\sqrt{N}$, achieving a coherent noise level at the scale of a single pixel of typically 1 nm, revealing fine neuronal processes of representative width of 1  μm with an SNR of around 5, when an air MO (20×, 0.8 NA) is used. These results show that this P-DHM approach has a strong capacity for coherent noise reduction that certainly goes beyond the performances presented, paving thus the way to coherent-noise-free OPD images. A more complete study of these coherent noise reduction capabilities and limitations (increasing the number of recorded holograms and changing the wavelength sampling) is, however, beyond the scope of this letter. Therefore, P-DHM represents a very promising noninvasive technique to study neuronal connectivity, particularly cellular processes taking place over extended periods of time (several hours to several weeks), such as network development including neurite outgrowth, elongation, and branching.
