# Polychromatic digital holographic microscopy: a quasicoherent-noise-free imaging technique to explore the connectivity of living neuronal networks (Publisher’s Note)

**DOI:** 10.1117/1.NPh.8.1.019801

**Published:** 2021-01-29

**Authors:** Céline Larivière-Loiselle, Erik Bélanger, Pierre Marquet

**Affiliations:** a Université Laval, Centre de recherche CERVO, Québec, Canada; b Université Laval, Département de physique, de génie physique et d’optique, Faculté des sciences et de génie, Québec, Canada; c Université Laval, Centre d’optique, photonique et laser, Québec, Canada; d Université Laval, Département de psychiatrie et neurosciences, Faculté de médecine, Québec, Canada

## Abstract

A section heading and paragraph missing from the originally published article are restored; a reference citation is repositioned.

This article [*Neurophotonics* **7**(4), 040501 (2020) doi: 10.1117/1.NPh.7.4.040501] was originally published on 16 October 2020 with a subsection missing from Sec. 3, Results and Discussion: “Disperson of the Observed Cells,” Sec. 3.3, containing a single paragraph, was inadvertently excised from the galley proof in the course of correction, causing loss of information for the reader.

The missing section heading and paragraph were restored on 20 January 2021. As part of that correction, a reference citation that had been erroneously repositioned in Sec. 2.2 (Ref. 33) was restored to Sec. 3.3.

