# Supplementary material for: Polychromatic digital holographic microscopy: a quasicoherent-noise-free imaging technique to explore the connectivity of living neuronal networks
Source: Neurophotonics. 2020 Oct 16;7(4):040501. doi: 10.1117/1.NPh.7.4.040501 (PMC7567399; doi:10.1117/1.NPh.7.4.040501)
Supplement: Supplementary file 1 [file NPh_007_040501_SD001.docx]

Supplementary Material

# Rat Neuronal Cell Culture

Primary rat neurons were obtained from dissected cortices from postnatal P0 rats (Sprague Dawley), as described by Lévesque et al^26^. Cells were deposited at an average density of 1.5 to 2.0 million cells∕cm^2^ on 18-mm-diameter round borosilicate coated coverslips (GG18-1.5-PDL, Neuvitro). Neurons were then cultured for two weeks in Neurobasal medium (LS21103049, Gibco™) supplemented with B27 (LS17504-044, Gibco™) and Glutamax (LS35050-061, Gibco™) and maintained at 37°C in an atmosphere humidified with 95% air/5% CO_2_, to obtain mature neurons forming a functional network. Half of the culture medium was changed every 3 days to ensure good culture conditions for the neurons. Neurons were imaged at 20 days of age.

# Methodology

Firstly, according to experimental observations, the emitted power by the supercontinuum white light laser and subsequently transmitted by the acousto-optic tunable filter fluctuates from single to double by changing wavelength over the 350-nm used bandwidth. Furthermore, the quantum efficiency of the sensor of the camera changes by a factor of 5 between 500 nm and 850 nm. Therefore, the hologram intensity detected by the camera significantly varies as a function of the wavelength. Since the ratio between the intensity of the object and reference arms in the microscope is not significantly modified by a wavelength change, adjusting the camera exposure time therefore makes it possible to record each of these holograms of different wavelengths in an optimal and as similar as possible manner. Typically, for each wavelength, the exposure time has been adjusted so that the most prominent hologram fringes usually located in its center approach but without reaching the maximum permissible gray level value.

Secondly, at each wavelength, the numerical refocusing performed during the hologram reconstruction was done manually. More specifically, the reconstruction distance was adjusted offline following a criterion minimizing the contrast of the amplitude image using a proprietary software (Koala, Lyncée Tec).

Thirdly, the procedure that was implemented to numerically correct the aberrations is a two-step process, applied for each wavelength, involving two phase masks (unit-amplitude complex numbers, see Eq. (5), Ref.^32^). The first phase mask is applied to the filtered hologram (corresponding to the virtual image frequency) before the wavefront propagation within Fresnel approximation using a convolution formulation. It has been calculated using a wavelength-matched reference conjugated hologram acquired in a blank situation (*i.e.*, a microscope without biological sample or imaging chamber) prior to the experiment. This phase mask corrects at each of the wavelengths for phase aberrations including the tilt aberration due to the off-axis geometry. The application of the second phase mask, this time in the image plane, corrects residual aberrations resulting from the fact that the blank situation is not strictly identical to the experimental conditions when biological specimens are present. This phase mask is numerically determined with an automatic procedure based on the determination of the phase reconstruction parameters from line profiles or 2D regions, located around the sample in zones that are assumed to be flat and used as reference surfaces^29^. The background of these reconstructed quantitative-phase images is then further flattened using illumination functions^26^.

# Polychromatic Digital Holographic Microscopy Denoising


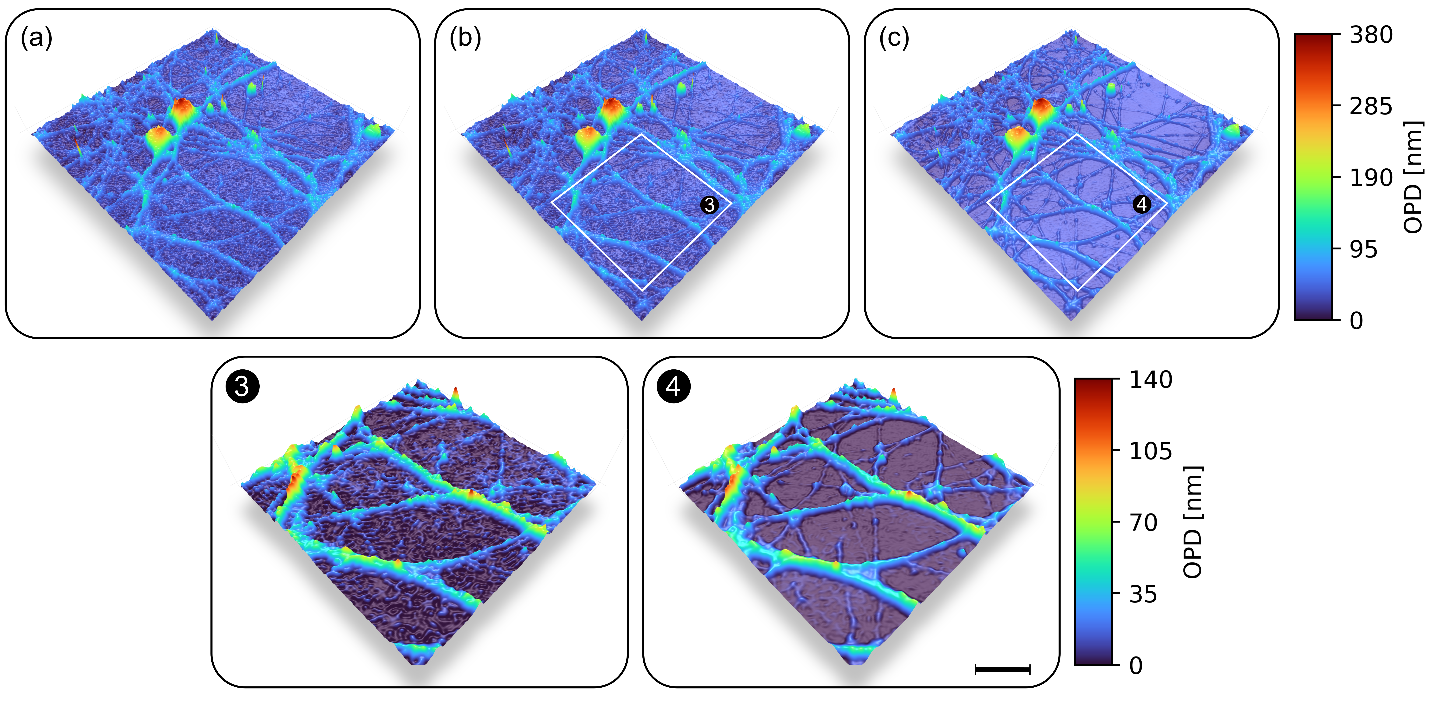


**Fig. S1** 3D rendering of the (a) single-shot OPD image acquired with 540 nm illumination, (b) control image, and (c) P-DHM image. (3)-(4) Enlarged neuronal process area of the control and P-DHM images respectively, with corresponding rescaled color bar on the right. Scale bar indicates 10 μm.

# Dispersion of the Observed Cells


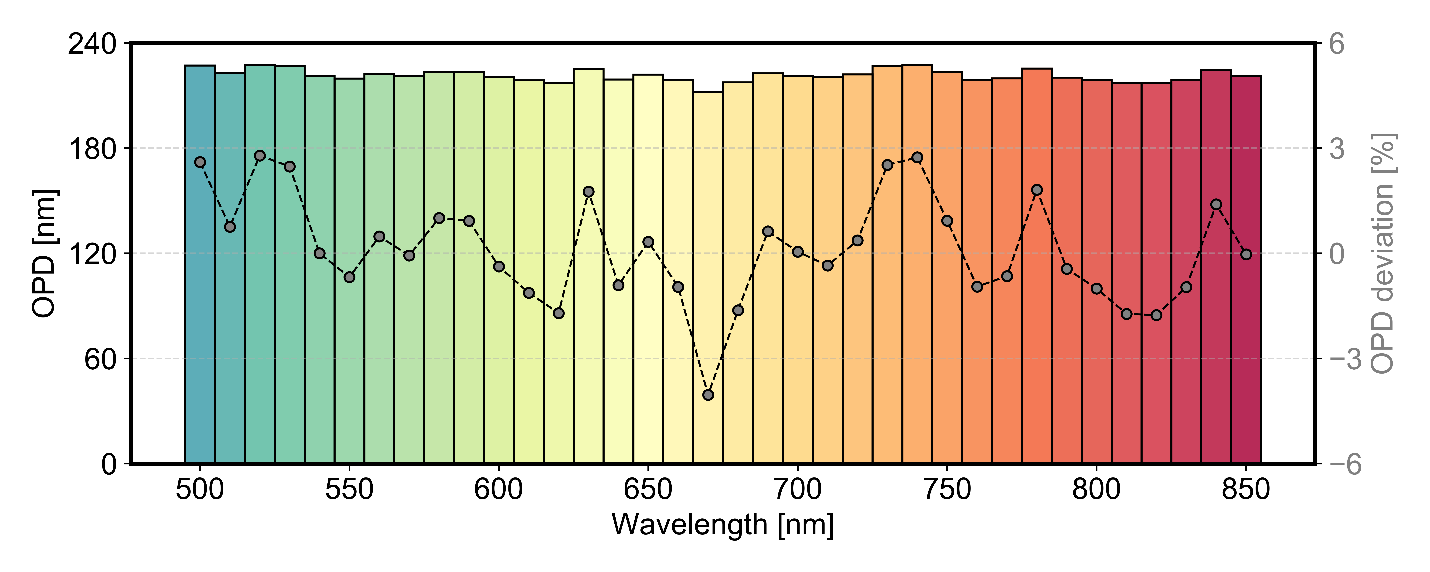


**Fig. S2** Mean OPD signal (left) and its deviation relative to the 540-nm OPD value expressed in percentage (right) measured on the left-hand neuronal cell body displayed in Fig. 2(c) as a function of the illumination wavelength used in P-DHM.
